# Life-limiting health conditions, treatment and subsequent mortality associated with differences in health service use in people with severe mental illness: an analysis of the Northern Ireland population using administrative data sources.

**DOI:** 10.23889/ijpds.v7i3.2055

**Published:** 2022-08-25

**Authors:** Rachel McCarter, Michael Rosato, Gerry Leavey

**Affiliations:** 1 Ulster University

## Objectives

To examine differences in hospital admissions for people with severe mental illness (SMI), physical health multimorbidity and mortality compared to the general hospital population without SMI.

## Approach

This study linked electronically captured routine administrative data from Northern Ireland (from 2013-2021): this comprised hospital records (including diagnostics codes); community prescribed medications; standard indicators of social deprivation (NIMDM); mortality data (General Register Office); and associated demographic data. The population included for analysis comprised 1,153,447 persons registered with a general practice at 01/01/2010 who subsequently attended a hospital setting. SMI was classified as a binary variable.

## Results

The population comprised 933,816 hospital patients aged 20plus at 2010, 414,887 (44.4%) males and 518,929 (55.5%) females – with 13,920 (1.5%) recording SMI: 6492 (46.6%) males and 7433 (53.4%) females between 2010 and 2021. Of these 7620 (54.7%) recorded schizophrenia; 4138 (29.7%) bi-polar disorder; and 2162 (15.5%) personality disorder. Those in the most deprived and those in urban areas were more likely to record SMI than their respective least deprived or rural peers (OR=2.38:95%CI=2.39-2.59 and OR=2.04:1.94-2.14). Increasing multimorbidity was associated with increasing SMI diagnosis: after adjustment for age, sex and year of exposure those with 5plus morbid states recorded OR=2.85(2.44-3.33) and OR=3.74(3.26-4.29) for 2012 and 2020 respectively. Finally, in models adjusted for age, sex and multimorbidity, those with SMI were more likely to die than their non-SMI peers: OR=2.09(1.92-2.29) for the 2012 group.

## Conclusion

Physical health multimorbidity and mortality increased in SMI compared to the non-SMI diagnosed group. We suggest a range of morbidities associated with SMI can drive excess mortality. This study highlights the importance to examine the extent of underdiagnosis of the causes of mortality in SMI presenting insights into suitable interventions.

## Acknowledgement

The authors would like to acknowledge the help provided by the staff of the Honest Broker Service (HBS) within the Business Services Organisation Northern Ireland (BSO). The HBS is funded by BSO and the Department of Health (DoH). The authors alone are responsible for the interpretation of the data and any views or opinions presented are solely those of the author and do not necessarily represent those of the BSO.

